# miR-25 promotes invasion of human non-small cell lung cancer via CDH1

**DOI:** 10.1080/21655979.2019.1632668

**Published:** 2019-07-06

**Authors:** Bing Liu, Xuerong Sun

**Affiliations:** a Department of Thoracic surgery, the central hospital of Linyi, Yishui, Shandong, China; b Department of Clinical Lab, Qingdao Women and Children’s Hospital, Qingdao, Shandong, China

**Keywords:** Non-small cell lung cancer, lymph node metastasis, miR-25, CDH1

## Abstract

MicroRNA-25 (miR-25) has been reported to be overexpressed in numerous human tumors and plays a key role in tumor promotor. However, there are few reports about miR-25 expression and function in non-small cell lung cancer (NSCLC). In this study, we investigated the biological role of miR-25 in NSCLC and its underlying molecular mechanisms. We found that the upregulation of miR-25 was correlated with lymph node metastasis and TNM stage in 113 NSCLC patients. Moreover, the up-regulation of miR-25 was associated with poor survival of NSCLC patients and might be used as an independent prognostic factor. Moreover, forced expression of miR-25 enhanced H358 and Calu-1 cell migration and invasion, but not apoptosis and proliferation *in vitro*. Elevation of invasion and metastasis by miR-25 directly and significantly correlated with inactivation of CDH1 expression. Therefore, patients with up-regulated miR-25 are prone to lymph node metastasis and thus have a poor prognosis.

## Graphical Abstract

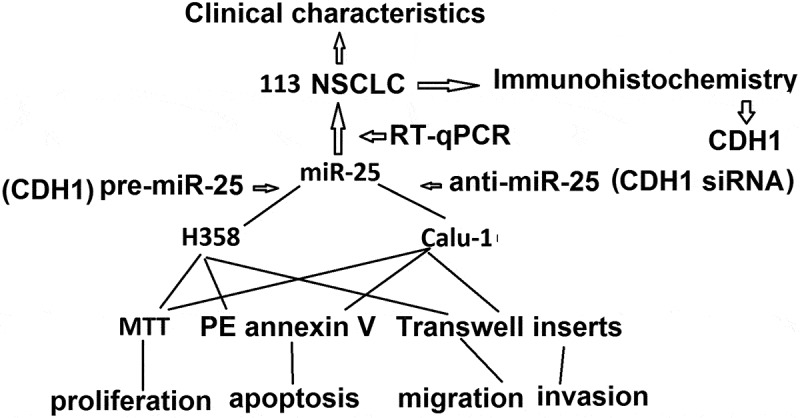

## Introduction

Lung cancer is the leading cause of tumor-related mortality worldwide. Non-small cell lung cancer (NSCLC) accounts for 80–85% of all lung cancer cases, and its 5-year survival rate is poor, and even for patients with early-stage disease who undergo curatively intended surgery, the postoperative recurrence rate is high compared to other types of cancer [1]. Tumor metastasis is primarily responsible for NSCLC mortality, yet the molecular mechanism of metastatic dissemination remains unclear.

Recent evidences suggest that miRNAs play an important role in tumor metastasis [2–5]. miRNAs are small ncRNAs around 22 nucleotides long and execute their post-transcriptional regulatory effects by binding to specific sites known as miRNA response elements (MREs) on their target transcripts, resulting in either transcript degradation or translational inhibition [6,7]. Various studies have demonstrated tissue – and cell-type specific expression of miRNAs, which could exhibit either tumor suppressive or oncogenic effects in a context-dependent manner [8,9].

MiR-25 is a member of the miR-106b~25 cluster, which includes miR-106b, miR-93, and miR-25, that is located within intron 13 of the minichromosome maintenance protein7 (MCM7) gene on chromosome 7q22.1 [10]. Previous studies have reported that miR-25 plays an important role in many biological processes. The expression of miR-25-3p was significantly increased in the plasma of thyroid papillary carcinoma, as compared with patients with benign tumors or healthy individuals [11]. miR-25 expression was higher in ovarian epithelial tissue, gastric cancer, lung adenocarcinoma, and many other tumors, and miR-25 expression levels were also closely related to tumor stage and lymph node metastasis [12–15]. In the present study, we investigated whether the relative expression of miR-25 between tumor and normal tissues is correlated with lymph node metastasis in NSCLC patients and the mechanism by which miR-25 promotes NSCLC metastasis.

## Materials and methods

### Patients

NSCLC tumor tissues (T) and normal adjacent tissues (NAT) were retrospectively selected from 113 NSCLC patients who underwent surgery as their first and only treatment between 2005 and 2013 at the central hospital of Linyi, Shandong, China. All tissue samples were snap frozen in liquid nitrogen immediately after surgery and stored at −80°C until the extraction of miRNA. For all the samples, clinicopathologic information was available. Corresponding formalin-fixed and paraffin-embedded (FFPE) tissues were available from 72 of 113 samples. Of 113 patients, 67 patients underwent surgery from 2005 to 2010 whose 5-year follow-up information was all available: median age was 56 years (range, 42–73); 88.3% were males; and median follow-up months were 43 (7–78 months). Approval for this study was obtained from the Institutional Review Board of the central hospital of Linyi. Signed informed consent was also obtained.

### miRNA and mRNA quantification

Total miRNA was isolated from frozen tissues using a mirVana miRNA isolation kit (Ambion). Total RNA was isolated from cultured cells using TRIzol reagent (Invitrogen). For RT-PCR, reverse transcription of total RNA was performed by Reverse Transcription System (Promega) or TaqMan Reverse Transcription system (Applied Biosystems, UK) according to the manufacturer’s protocol. The qPCR was carried out using LightCycler® 480 System (Roche, Mannheim, Germany) with the universal SYBR Green PCR master mix (Roche) by using *β-actin* as an internal control. The levels of CDH1 transcript were measured by forward primer, 5ʹ-AGAACGCATTGCCACATACA-3ʹ, and reverse primer, 5ʹ-TGCTTAACCCCTCACCTTGA-3ʹ. b-Actin was used as internal control and amplified with forward primer, 5ʹ-CGCGAGAAGATGACCCAGATC-3ʹ, and reverse primer, 5ʹ-TGGTACGGCCAGAGGCG-3ʹ. All results from three independent experiments are presented as mean ± s.e.m. (n = 3).

Expression of mature miR-25 was analyzed using the TaqMan® MicroRNA Assays (Applied Biosystems). Expression of RNU6B (Applied Biosystems) was used as an endogenous control. miR-25 expression was measured relative to RNU6b (internal control) and quantified by the relative Ct method (2^ΔΔCt^). All the results are from three independent experiments done in duplicate. Two commercially avoidable normal brain cDNA libraries were used as normal control (Invitrogen, Grand Island, NY; Biochain, Hayward, CA). The TaqMan qPCR was carried out using LightCycler® 480 System (Roche) with the TaqMan universal PCR master mix (Applied Biosystems). The 75th percentiles of 2^ΔΔCt^ were used as the cutoff point for patients with high and low levels of miR-25. All results from three independent experiments were performed in duplicate are presented as mean ± s.e.m. (n = 3).

### Immuno his to chemistry

Tissue sections were dewaxed with xylene and rehydrated through gradient ethanol into water. For antigen retrieval, sections were heated in citrate buffer (pH 6.0) for 10 min at 95°C in a microwave oven. After cooling to room temperature, the sections were then digested with 0.05% trypsin for 10 min at 37°C. Endogenous peroxidase activity was quenched with 0.3% H_2_O_2_ in methanol for 30 min at room temperature. After PBS washes, nonspecific antibody binding was blocked by preincubating slides with 10% normal goat non-immune serum at 37° for 30 min. After blotting off the blocking serum, sections were incubated with primary antibody against CDH1 (1:200 dilution) at 4° overnight. After PBS washes again, sections were incubated with biotinylated secondary antibody at 1:200 dilution for 30 min at room temperature. After incubating with Vectastain ABC reagent (Vector Laboratories, Inc., Burlingame, CA) for 30 min at room temperature, the sections were developed with diaminobenzidine (Sigma-Aldrich). Sections were washed in running tap water and lightly counterstained with hematoxylin, followed by dehydration and coverslip mounting. Negative controls were obtained by omitting the primary antibody. Expression CDH1 were evaluated as described previously. The percentage of positive tumor cells was determined semi-quantitatively by assessing the entire tumor section. Each sample was assigned to one of the following categories: 0 (0–4%), 1 (5–24%), 2 (25–49%), 3 (50–74%), or 4 (75–100%). The intensity of immunostaining was determined as 0 (negative), 1+ (weak), 2+ (moderate), or 3+ (strong). A final immunoreactive score between 0 and 12 was calculated by multiplying the percentage of positive cells with the staining intensity score. The two scores were then multiplied to produce a weighted score for each sample, the expression was considered positive when the score was >2. All slides were blindly evaluated for immunostaining without any knowledge of the clinical outcome of other clinical or pathological data.

### Western blot analysis

Cells were harvested and washed with PBS twice, disrupted in IP buffer (Thermo) and centrifuged at 12,000 × g for 20 min. Protein (50 μg) from the supernatant fraction (quantified by the BCA Protein Assay Kit, Thermo) was subjected to SDS-PAGE and transferred to a PVDF membrane (Millipore). Membranes were blocked with 5% non-fat milk for 1 h at room temperature and then incubated with the anti-CDH1, followed by the corresponding HRP-conjugated anti-mouse or anti-rabbit secondary antibody. Protein bands were visualized by the Western lightening plus-ECL kit (Pierce).

### Cell culture

All NSCLC lines (H2087, HCC44, Calu-1, H358, H1993) used in this study were obtained from the Hamon Cancer Center Collection (University of Texas Southwestern Medical Center), maintained in RPMI-1640 (Life Technologies), and supplemented with 5% FBS, penicillin (100 units/ml), and streptomycin (100 μg/ml) at 37°C in a humidified atmosphere containing 5% CO_2_. All cell lines have been DNA fingerprinted using the PowerPlex 1.2 kit (Promega) and are mycoplasma free using the e-Myco kit (Boca Scientific).

### Transfection

The miR-25 precursor (pre-miR-25), miR-25 inhibitor (anti-miR-25), and FAM-labeled pre-miR and anti-miR negative control (pre-miR-nc, anti-miR-nc) were purchased from Ambion and transfected at a final concentration of 30 nM with Lipofectamine 2000 (Invitrogen) following the manufacturer’s instruction. siRNA against CDH1 and scrambled siRNA negative control were purchased from Invitrogen and transfected at a final concentration of 40 nM.

### Cell proliferation, apoptosis, migration, and invasion assay

To measure cell proliferation, cells were plated at a density of 1 × 10^3^ cells per well onto 96-well plates at day 0 (24 h after pre-miR-25 or anti-miR-25 transfection). At days 1, 3 and 5, cell viability was measured using MTT as the manufacturer's instruction.

Apoptosis was tested by using PE annexin V apoptosis detection kit (BD Pharminge). Forty-eight hours after transfection, 4 × 10^5^ cells were incubated with annexin V-PE/7-AAD for 15 min at room temperature, and then analyzed by Coulter Epics XL flow cytometry (Beckman).

To measure cell migration and invasion,12 h after transfection, cells were starved with serum-free medium for 12 h, then planted into the Transwell inserts in 24-well plate with 8.0-mm pores (Corning). 100 ml (5 × 10^5^ cells/ml) cells were seeded atop inserts membranes uncoated or coated with Matrigel (BD Biosciences). Cells were incubated with serum-free medium and translocated toward medium with 10% FBS for 24–36 h. Membranes were stained by 0.5% crystal violet dissolved in 100% methanol. pCMV6-CDH1, which contains CDH1 open reading frame was purchased from OriGene Technologies.

To inhibit miR-25 expression, Calu-1 cells were plated in 24-well plate and transfected with anti-miR-25; To increase miR-25 expression, H358 cells were plated in 24-well plate and transfected with pre-miR-25; To restore the miR-25-resistant CDH1 expression, H358 cells were plated in 24-well plate and transfected with pre-miR-25 and pCMV6-CDH1. To inhibit the miR-25-dependent CDH1 expression, Calu-1 cells were plated in 24-well plate and transfected with siRNA against CDH1 and scrambled siRNA negative control. Twenty-four hours after transfection, cells were digested and subjected to the migration and invasion assay as discussed above. The average number of invasive/migrative cells was counted in six random high-power fields (×400).

### Statistical analysis

The unpaired Student’s *t* test was used with *p <* 0.05 considered significant. Results were displayed as mean±S.E. from at least triplicate experiments for each group. All statistical analyses were performed with SPSS 22.0 software. Three times of each experiment were performed.

## Results

### Expression of miR-25 is correlated with lymph node metastasis and TNM stage in NSCLC patients

**Figure 1.**
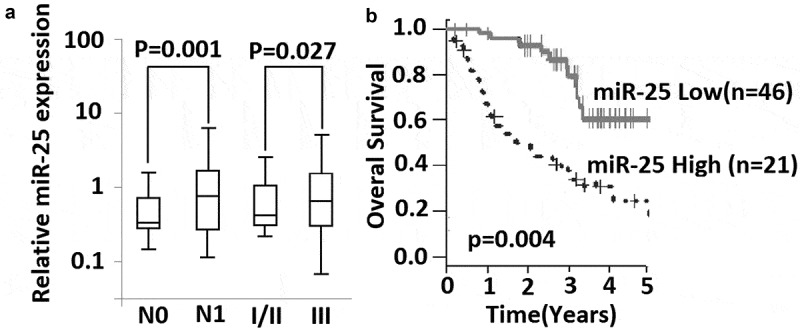
Expression of miR-25 is associated with lymph node metastasis and survival of NSCLC patients. (a) relative expression of miR-25 was compared between lymph node metastasis (*N1*) and without lymph node metastasis (*N0*), and between patients in stage I-II and patients in stage III. Mann–Whitney *U* test was used. Box and whisker show median value with 10–90% percentile. (b) 67 NSCLC patients with their following information available were classified into high expression group (*n*= 21) and low expression group (*n*= 46) by the 75th percentiles of 2^ΔΔCt^. Shown is Kaplan-Meier overall survival curves of two group patients, Log-rank test was used.

The expression of miR-25 in NSCLC tissues was detected by qRT-PCR, and the relationship between the relative expression of miR-25 and clinicopathologic information of NSCLC patients was analyzed. The relative expression of miR-25 significantly increased in patients with lymph node metastasis compared with patients without lymph node metastasis (p < 0.001) (Figure 1a and Table 1). The relative expression of miR-25 in NSCLC samples was also found to be correlated with the TNM stage of patients (p = 0.017) (Figure 1a and Table 1). No significant association was found between miR-25 expression and other clinical characteristics (Table 1).

**Table 1.** Association between miR-25 expression and clinicopathologic information of NSCLC patients.

Clinical parameters		miR-25 expression		
	Total (*n* = 113)	Low	High	*p* Value
Sex				0.46
Female	24	18	6	
Male	89	65	24	
Age				0.76
≤60 years	50		14	
> 60 years	63		16	
Smoking status				0.14
Never	44		12	
Previous/current	69		18	
Histological type				0.48
Adenocarcinoma	92		22	
Squamous carcinoma	21		8	
TNM Stage				0.027
I/II	85		14	
III	28		16	
Lymph node metastasis				0.001
N1	83		12	
N0	30		18	
Tumor size (cm)				0.126
≤2	37		11	
>2	76		19	
Differentiation				0.24
well+ moderate	70		20	
poor	43		10

### High level of miR-25 is correlated with poor survival of NSCLC patients

The potential of miR-25 as a prognosis marker was further investigated in 67 patients. These patients underwent NSCLC resection from 2005 to 2010, and their 5-year follow-up information was available for each patient. This subgroup of patients had similar clinical characteristics as compared with a total of 113 patients. Kaplan-Meier survival estimate showed that the patients with high miR-25 expression had shorter survival compared with the patients with low miR-25 expression. The difference in the prognosis of these two groups was statistically significant (p = 0.004;log-rank test) (Figure 1a). Next, univariate Cox analysis was performed to determine whether overall survival is associated with other prognostic factors, including differentiation grade, N, and TNM stages. We found that overall survival was correlated with miR-25 expression and N and TNM stages, but not differentiation grade (Table 1). Furthermore, multivariate Cox proportional hazard regression analysis was performed using all three variables and showed that high miR-25 expression was an unfavorable prognostic factor (p = 0.012; risk ratio 1.84; 95% confidence interval, 0.98–4.14) along with TNM stage (p = 0.043; risk ratio 3.14; 95% confidence interval, 1.42–4.94) (Table 2).

**Table 2.** Overall survival of NSCLC patients in relation to clinicopathologic characteristics and miR-25 expression.

	Univariate analysis		Multivariate analysis	
Variable (*n*= 67)	Hazard ratio (95% CI)	*p*	Hazard ratio (95% CI)	*p*
Differentiation (well+ moderate/poor)	2.35 (1.07–5.14)	0.085		
N (N1/N0)	2.75 (1.27–5.546)	0.006		
TNM stage (I+ II/III)	2.98(1.26–4.17)	0.023	3.14(1.42–4.94)	0.043
miR-25 (low/high)	1.93(0.98–4.14)	0.004	1.84(1.07–5.74)	0.012

### miR-25 modulates migration and invasion but not proliferation in NSCLC cells

**Figure 2.**
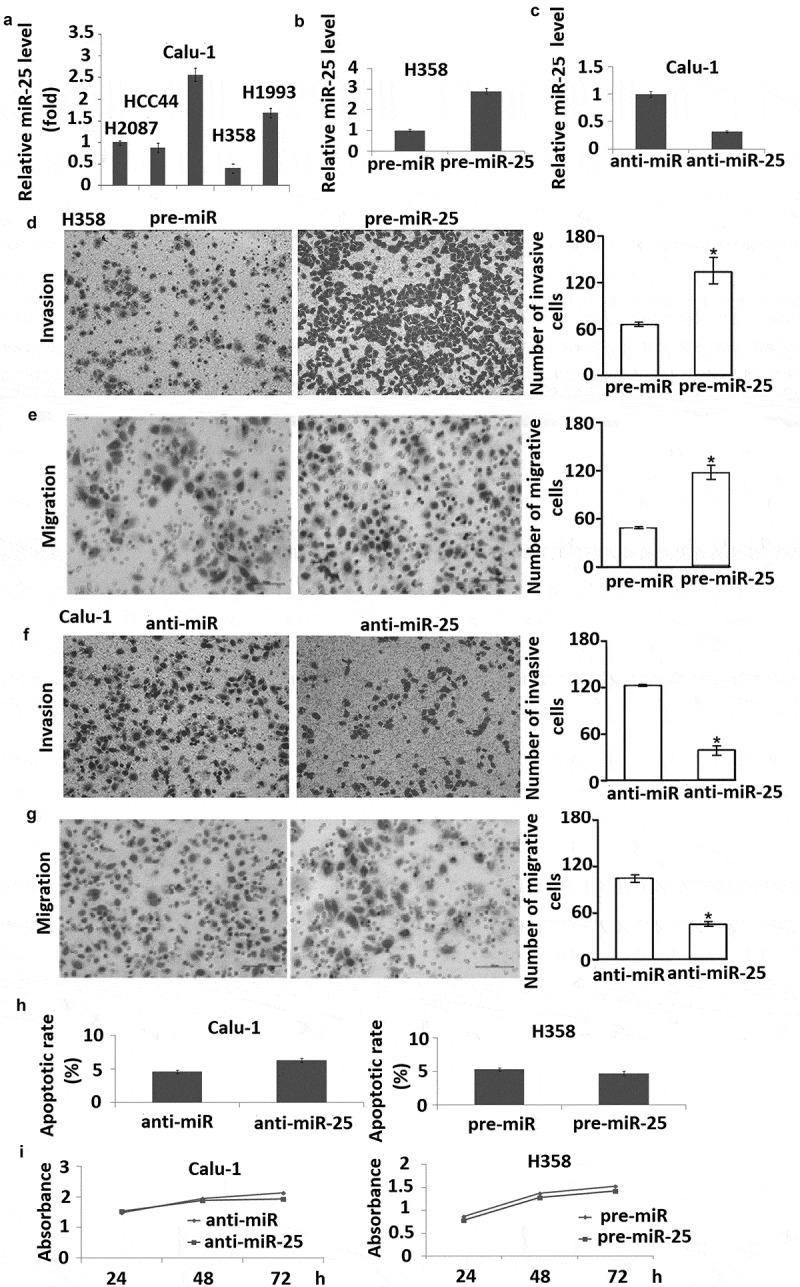
miR-25 modulates cell migration and invasion in NSCLC cells. (a) Expression of miR-25 in H2087, HCC44, Calu-1, H358, and H1993 cells were detected by qRT-PCR, RNU6B served as internal control; (b) H358 cells transfected with pre-miR-25 and pre-miR for 48 h were harvested for detection of miR-25 expression by qRT-PCR, RNU6B served as internal control. (c) Calu-1 cells transfected with anti-miR-25 and anti-miR for 48 h were harvested for detection of miR-25 expression by qRT-PCR, RNU6B served as internal control. H358 cells were transfected with 30 nM pre-miR-25 or pre-miR for 24 h, cell invasion (d) and migration (e) was detected. Calu-1 cells transfected with anti-miR-25 and anti-miR for 24 h, cell invasion (f) and migration (g) were detected. (h) H358 was transfected with 30 nM pre-miR and pre-miR-25, and Calu-1 was transfected with anti-miR and anti-25. Forty-eight hours after transfection, cells were collected for apoptosis analysis by annexin V staining and flow cytometry. (i) H358 was transfected with 30 nM pre-miR and pre-miR-25, and Calu-1 was transfected with anti-miR and anti-miR-25. Twenty-four hours after transfection, cell viability was detected using MTT at days 1, 3, and 5 after seeded; the proliferation curve was generated based on the absorbance and times. *Columns*, mean for three experiments, *bars*, S.E.; * *p*< 0.01.

To investigate the mechanism by which miR-25 promotes lymph node metastasis, we examined the role of miR-25 in **NSCLC** cell migration and invasion. miR-25 expression was detected by qRT-PCR in H2087, HCC44, Calu-1, H358, H1993 cells, showing that H358 cells have the lowest miR-25 expression, and Calu-1 has the highest miR-25 expression (Figure 2a). So we used H358 and Calu-1 cells for further study.

The expression of miR-25 was dramatically increased by transfection of pre-miR-25 into H358 cells and decreased by transfection of anti-miR-25 into Calu-1 cells (Figure 2b,c). It was found that overexpression of miR-25 markedly enhanced migration of H358 cells compared with negative control (Figure 2d). Conversely, the inhibition of miR-25 expression led to a considerable decrease in the migration of Calu-1 cells compared with the negative control (Figure2e).

Next, we examined the effect of miR-25 on cell invasion across an extracellular matrix and showed that in H358 cells, overexpression of miR-25 markedly enhanced the potential of invasion compared with the control (Figure 2f). And knock-down of miR-25 in Calu-1 cells inhibited invasion compared with the control (Figure 2g). However, overexpression of miR-25 in H358 cells and knock-down of miR-25 in Calu-1 cells had no significant effect on cell apoptosis and proliferation (Figure 2h,i). These results demonstrated that miR-25 promotes NSCLC cell migration and invasion but has no effect on cell apoptosis and proliferation.

### Expression of CDH1 is inversely regulated by miR-25 in NSCLC

**Figure 3.**
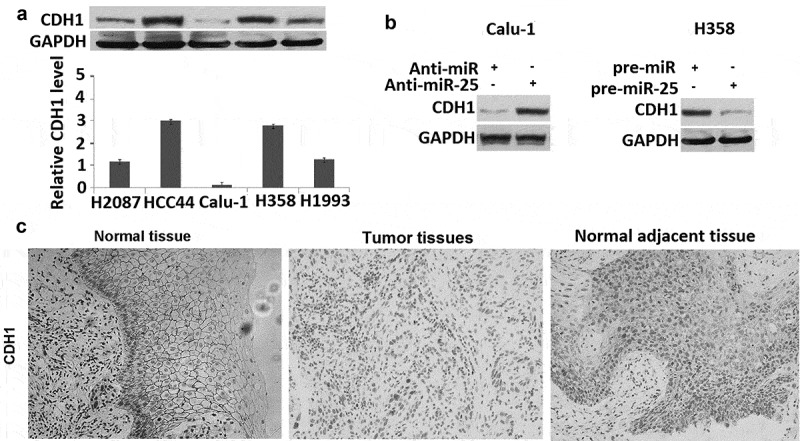
miR-25 regulates the expression of CDH1. (a) The cell lysates of H2087, HCC44, Calu-1, H358, and H1993 cells were prepared for Western blotting using antibody against CDH1; GAPDH was used as a loading control. (b) Calu-1 cells were transfected with 30 nM anti-miR and anti-miR-25, cells were collected for Western blotting using antibody against human CDH1 and GAPDH was used as a loading control. H358 cells were transfected with 30 nM pre-miR and pre-miR-25, cells were collected for Western blotting using antibody against human CDH1 and GAPDH was used as a loading control. (c) expression of CDH1 in normal tissues, tumor tissues, and tumor-adjacent tissues was examined by IHC.

The activity of miR-25 to promote cell migration and invasion might be due to its ability to regulate the expression of genes contributed to metastatic dissemination. To determine whether CDH1 was the target of miR-25, the relationship between miR-25 expression and CDH1 level was analyzed. CDH1 was detected in H2087, HCC44, Calu-1, H358, H1993 cells, showing that H358 cells have the highest miR-25 expression, and Calu-1 has the lowest miR-25 expression (Figure 3a), and there was a strong inverse relationship between miR-25 expression and CDH1 protein level in five **NSCLC** cell lines (Figure 2a). Moreover, the overexpression of miR-25 could significantly down-regulate the level of CDH1 protein in H358 cells. Conversely, the level of CDH1 protein was markedly up-regulated by suppressing the miR-25 expression in Calu-1 cells transfected with anti-miR-25 (Figure 3b).

To further determine the relationship between CDH1 and miR-25 in **NSCLC** tumor tissues, the level of CDH1 protein was measured by IHC in 72 **NSCLC** samples for which the corresponding **NSCLC** tumor tissues, and normal adjacent tissues were available (Figure 3c). It was found that the level of CDH1 was markedly lower in tumor tissues than that in normal adjacent tissues: 34 of 72 tumor tissues were scored as negative. Moreover, 64% (25 of 39) tumor tissues with high miR-25 levels showed negative CDH1 expression; whereas only 36.3% (12 of 33) tumor tissues of low miR-25 level were CDH1 negative (Table 3). These results indicated that CDH1 is a target of miR-25 in **NSCLC** cells.

**Table 3.** Relation between miR-25 and CDH1 expression in 72 NSCLC.

	CDH1 negative	CDH1 positive	Total	*p* Value
miR-25 low	12	21	33	
miR-25 high	25	14	39	
Total	37	35	72	0.014

### miR-25 mediated invasion and migration in NSCLC cells by CDH1 regulation

CDH1 has been proven to play a critical role in cell migration and invasion. We confirmed above that targeting miR-25 markedly increased migration and invasion of Calu-1 cells, followed by increased CDH1 expression. In addition, miR-25 overexpression markedly decreased migration and invasion of H358 cells, followed by decreased CDH1 expression.

**Figure 4.**
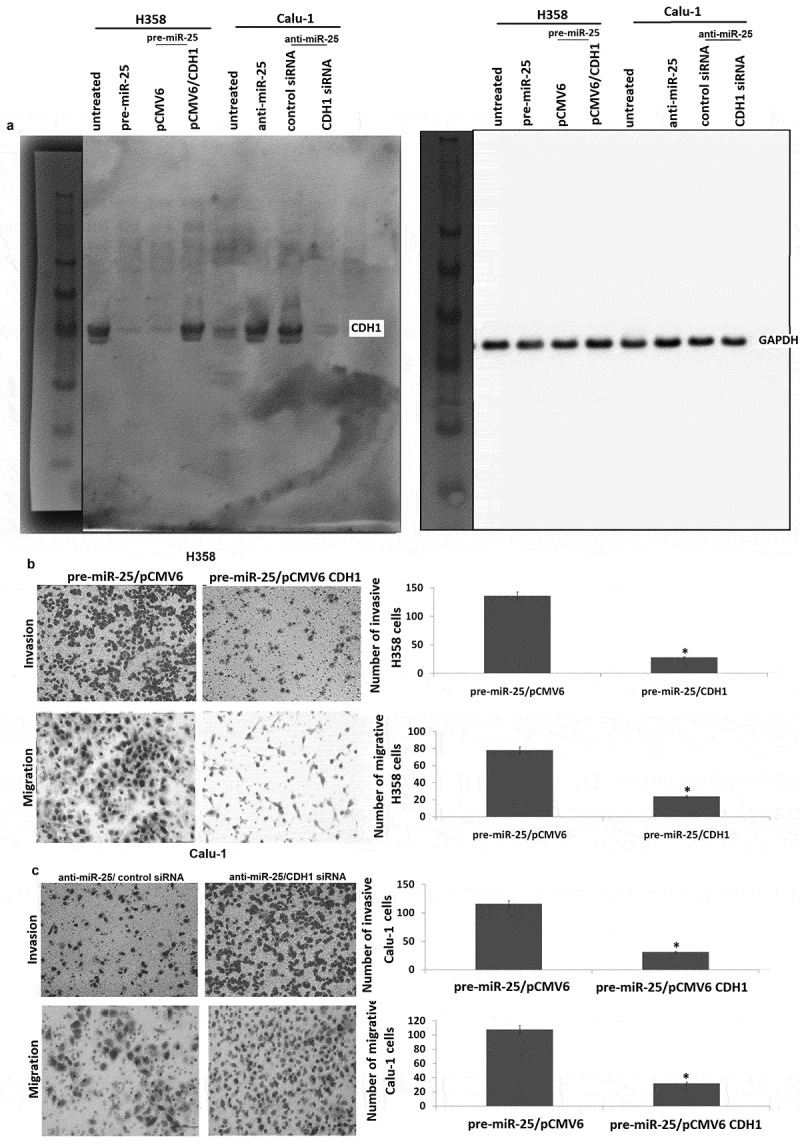
CDH1 plays a critical role in miR-25-mediated invasion and migration in NSCLC cells. (a) H358 cells were co-transfected with pre-miR or pre-miR-25 and pCMV6 or pCMV6-CDH1 for 24 h, Calu-1 cells were co-transfected with anti-miR or anti-miR-25 and control siRNA or CDH1 siRNA for 24 h, expression of CDH1 was detected by Western blot assay. (b) H358 cells were co-transfected with pre-miR or pre-miR-25 and pCMV6 or pCMV6-CDH1 for 24 h, cell invasion and cell migration were detected. (c) Calu-1 cells were co-transfected with anti-miR or anti-miR-25 and control siRNA or CDH1 siRNA for 24 h, cell invasion and cell migration were detected. Cell lysates were prepared for Western blotting using antibody against CDH1 after transfection. *p < 0.05.

To demonstrate the contribution of CDH1 to the biological function of miR-25, we first examined whether re-expression of CDH1 has an effect on miR-25-induced cell migration and invasion in H358 cells. To test this, pre-miR-25 and pCMV6-CDH1 were co-transfected into H358 cells. The results showed that co-transfection of pre-miR-25 and pCMV6-CDH1 restored CDH1 expression in the pre-miR-25 transfected H358 cells (Figure 4a). Furthermore, constitutive expression or CDH1 abrogated the effect of miR-25 on cell invasion and migration (Figure 4b). We next determined whether targeting CDH1 could abrogate the effect of targeting miR-25 on cell invasion and migration. To test this, anti-miR-25 and CDH1 siRNA were co-transfected into Calu-1 cells. The results showed that co-transfection of anti-miR-25 and CDH1 siRNA reversed CDH1 expression in the anti-miR-25 transfected Calu-1 cells (Figure 4a). Furthermore, targeting CDH1 abrogated the effect of targeting miR-25 on cell invasion and migration (Figure 4b).

## Discussion

An ethnically diverse multicenter case–control study recruiting 221 NSCLC patients, 161 controls, and 56 patients with benign nodules from China and America reported that serum levels of miR-25 and other four miRNAs (miR-483-5p, miR-193a-3p, miR-214, and miR-7) were significantly elevated irrespective of ethnicity groups [16]. A clinical study enrolling 100 Chinese female non-smoking lung adenocarcinoma patients found that increased plasma miR-25 levels positively correlated with the mortality rate, advanced disease stage, regional and distant metastasis at diagnosis [17]. Another study has reported that significantly elevated serum miR-25 levels were negatively correlated with progression-free survival time [18]. Wu et al. have reported that miR-25 expression was elevated in the plasma of NSCLC patients and NSCLC cell lines [19]. Ding and Xiang et al. have reported that miR-25 was overexpressed in 31 NSCLC tissues compared with the corresponding normal tissues [20,21], which was consistent with our present findings. But, Wu, and Xiang et al. did not research the relationship between miR-25 expression and clinical pathology in NSCLC patients [19,21]. However, our study showed that the expression of miR-25 is significantly correlated with lymph node metastasis and TNM stage of NSCLC patients, which was consistent with Ding’s [20]. Cell motility and invasion are required for the dissemination of tumor cells from their primary location to lymph or blood vessels during metastasis. Several miRNAs were found to exhibit pro-metastatic (miR-10b, 21, and 373/520c) or anti-metastatic (miR-34b/c, 126, 148a, 206, and 335) activity [22–24]. Our data showed that miR-25 promotes cell migration and invasion, which is consistent with our tissue correlation study in which the level of miR-25 is associated with lymph node metastasis. Our results were also consistent with the previous findings that miR-25 promotes NSCLC cell migration and invasion *in vitro* [20,21]. Wu et al. [19] found that transfection of A549 and 95-D cells with a miR-25 inhibitor resulted in reduced cell proliferation and enhanced apoptosis. Xiang et al. [21] have also reported that miR-25 promoted A549 cell proliferation. These data indicated that targeting miR-25 inhibited NSCLC cell migration and invasion partly by promoting cell apoptosis. In our study, overexpression of miR-25 in H358 cells and targeting miR-25 in Calu-1 cells had no significant effect on cell apoptosis and proliferation, suggesting that targeting miR-25 inhibited NSCLC cell migration and invasion not via promoting cell apoptosis. These data indicated that the role of miR-25 in different NSCLC cells is different. Although targeting miR-25 inhibited A549 and Calu1 invasion in vitro in Ding’s study [20], they did not study whether miR-25 could affect cell apoptosis and proliferation, which is associated with cell invasion.

The biological function of miR-25 has been examined through the identification of miR-25 targets. Ding et al. demonstrated that miR-25 was demonstrated to activate the ERK signaling pathway by directly targeting KLF4, promoting cell migration and invasion [19]. Savita et al. reported that the overexpression of the miR-106b-25 cluster could directly suppress the ubiquitin ligase β-TRCP2 gene expression leading to decreased Snail degradation in H1299 non-small cell lung cancer cells [25]. Our current finding indicated that miR-25 represses the expression of CDH1 in NSCLC. It has been proven that loss of expression and/or abnormal function of CDH1 lead to loss of cell polarity and derangement of normal tissue architecture [26,27]. In most cancers with epithelial origins, CDH1-mediated cell-cell adhesion is lost concomitantly with the acquisition of an invasive phenotype, high tumor grade, and low patient survival [28,29]. Loss or reduction of CDH1 expression can be caused by a variety of mechanisms, such as somatic mutations, chromosomal deletions, proteolytic cleavage, and silencing of the CDH1 promoter [30]. Recent studies showed that the miR-200 and miR-205 families regulate ZEB1/EF1 and B2/SIP1 transcription repressors, which in turn regulate CDH1 expression [3]. Moreover, miR-9, which is directly bound and upregulated by MYC and MYCN in breast cancer cells, directly targets CDH1, leading to increased cell motility and invasiveness [31]. Our study revealed a novel mechanism by which CDH1 is regulated. We showed that CDH1 is directly targeted by miR-25 in NSCLC cell lines. In addition, we uncovered an inverse correlation between miR-25 and CDH1 in NSCLC tumor tissues and cell lines. Furthermore, restoration of CDH1 expression-inhibited cell invasion and migration induced by miR-25 overexpression, indicating that CDH1 functions as a mediator of miR-25 in cell migration and NSCLC progression. Nevertheless, we would like to note that the decrease in CDH1 expression was observed more commonly than the increase in miR-25 expression in NSCLC tumor tissues, suggesting that CDH1 is regulated by a mechanism in addition to miR-25 in NSCLC, which merits further investigation.

## Highlights

1. miR-25 is overexpressed in human NSCLC tissues.

2. Overexpression of miR-25 is correlated with lymph node metastasis and poor prognosis.

3. Targeting miR-25 inhibits migration and invasion in NSCLC cells via activating CDH1.

4. miR-25 did not affect apoptosis and proliferation in NSCLC cells in vitro.
